# An E3 ubiquitin-proteasome gene signature for predicting prognosis in patients with pancreatic cancer

**DOI:** 10.3389/fimmu.2023.1332626

**Published:** 2024-01-18

**Authors:** Taoyuan Yin, Jingjing Wen, Simiao Xu, Lin Chen, Zhenxiong Zhang, Shutao Pan, Min Zhou, Xingjun Guo, Min Wang, Jun Gong, Hang Zhang, Renyi Qin

**Affiliations:** ^1^ Department of Biliary-Pancreatic Surgery, Affiliated Tongji Hospital, Tongji Medical College, Huazhong University of Science and Technology, Wuhan, Hubei, China; ^2^ Department of Dermatology, Union Hospital, Tongji Medical College, Huazhong University of Science and Technology, Wuhan, Hubei, China; ^3^ Department of Endocrinology, Affiliated Tongji Hospital, Tongji Medical College, Huazhong University of Science and Technology, Wuhan, Hubei, China

**Keywords:** pancreatic cancer, ubiquitin-proteasome, prognosis, predictive model, gene signature

## Abstract

Pancreatic cancer is the seventh leading cause of cancer death worldwide, which is demonstrated with remarkable resistance to radiotherapy and chemotherapy. The identification of prognosis signature and novel prognostic markers will facilitate patient stratification and an individualized precision therapy strategy. In this study, TCGA-PAAD was used to screen prognostic E3 ubiquitin ligases and establish prognostic signatures, and GEO database was used to verify the accuracy of prognostic signatures. Functional analysis, *in vitro* experiments and clinical cohort studies were used to analyze the function and prognostic efficacy of the target gene. An E3 ligase-based signature of 9 genes and the nomogram were developed, and the signature was proved to accurately predict the prognosis of patients with pancreatic cancer. WDR37 might be the most prognostic E3 ubiquitin ligase in pancreatic cancer, and the clinical cohort analyses suggested a tumor‐suppressive role. The results of functional analysis and *in vitro* experiments indicated that WDR37 may promote the degradation of TCP1 complex to inhibit tumor and improve immune cell infiltration. The E3 ligase-based signature accurately predicted the prognosis of patients with pancreatic cancer, so it can be used as a decision-making tool to guide the treatment of patients with pancreatic cancer. At the same time, WDR37, the main gene in E3PMP signature, can be used as the most prognostic E3 ubiquitin ligase in the treatment of pancreatic cancer.

## 1 Introduction

Currently, pancreatic cancer is the seventh leading cause of cancer death worldwide, which is one of the most lethal cancers in the world (1). Pancreatic cancer is demonstrated with remarkable resistance to radiotherapy and chemotherapy due to genetic and epigenetic factors, along with a complex and desmoplastic tumor microenvironment (2). For these reasons, the identification of prognosis signature and novel prognostic markers for treatment approaches are imperative clinical need, which facilitates patient stratification and an individualized precision therapy strategy.

Ubiquitination is one of the most prevalent protein post-translational modifications, which participates in a number of important oncological processes, such as cell cycle, invasion and metastasis, tumor metabolism, and immune reactions (3). In the ubiquitination process, the Ub-activating (E1) and Ub-conjugating (E2) prepare ubiquitin for conjugation, while Ub-ligating (E3) transfers the ubiquitin to lysine residues on client proteins (4). Since E3 ubiquitin ligases provide substrate specificity for the conjugation between ubiquitin and target protein, protease-targeting chimeras (PROTAC) drugs represent a new avenue for cancer therapeutic research (5). Some E3 ubiquitin ligases have been reported to be associated with invasion and metastasis, drug resistance and the formation of immune microenvironment in pancreatic cancer, such as NEDD4, βTRCP1 and MDM2 (6–8). However, more than 800 proteins with E3 enzyme activity have been identified, and the importance of different E3 enzymes in pancreatic cancer is not clear (9). Hence, more systematical investigations on the role of E3 enzymes in pancreatic cancer are needed.

There is growing evidence that it is of great significance to construct the prognostic signature of pancreatic cancer based on the expression of E3 ubiquitin enzymes. In this research, the prognostic signature of nine E3 genes was constructed from TCGA data and verified in the GEO data set. Finally, the molecular mechanism and clinical effectiveness of the most important E3 enzyme in the prognostic signature were discussed and verified in the TCGA and clinical cohort.

## 2 Methods

### 2.1 Data collection and processing

The transcriptomic (HTSeq-FPKM) and clinical information of 178 tumor tissues and 332 normal pancreatic tissues were downloaded from TCGA-PAAD and GTEx databases as training sets for evaluations. Transcriptomic data was normalized using TPM format. The gene expression data and clinical information of GSE57495 and GSE62452 datasets were downloaded from GEO database as validation sets (10, 11). The combined datasets contained 128 pancreatic cancer samples with survival information. The batch effects from non-biotechnological bias were corrected by the “limma” package. A total of 898 E3 ubiquitin ligases were identified from iUUCD2.0 database (12).

### 2.2 Identification of prognostic genes and multivariate Cox regression analysis

In order to reduce the number of candidate genes, differential analysis and survival analysis were carried out. The expression of E3 ligases was extracted from the TCGA data set, and the differences in E3 ligase expression between tumor tissue and normal pancreatic tissue were calculated by the “limma” package. To show the differences in E3 ligase between tumor and normal pancreatic tissue, the “pheatmap” and “ggplot2” packages were used to draw the heat map and volcano plot. Univariate Cox regression analysis and log-rank test were used for survival analyses, where the “survminer” package was used to determine the best cut-off value for log-rank test. For survival analyses, patients with survival duration less than 30 days were excluded, since such deaths were generally attributed to complications. The candidate genes of differential analysis and survival analysis were identified using the “VennDiagram” package. The least absolute shrinkage and selection operator (LASSO) Cox regression algorithm was employed for feature selection using the “glmnet” package. The screening results were tested by hypothesis test and collinearity test, and finally a stepwise multivariate Cox regression analysis was carried to establish the prognostic signature. The risk score was expressed as the sum of each factor`s product with the regression coefficient.

### 2.3 Construction and verification of nomogram

According to the risk score, the nomogram of E3PMP prognostic signature of TCGA set was constructed to predict the overall survival of 1-year, 2-year and 5-year for patients with pancreatic cancer. Optimal cut-off value was identified with “survminer” package for stratification in survival. The calibration plot, Kaplan-Meier curve and the area (AUC) under receiver operating characteristics (ROC) curve analysis were performed to investigate the predictive value of the E3PMP prognostic signature. The ‘nomogramFormula’ package was used to obtain the calculation formula of the nomogram score, and the nomogram score of the verification set (GSE57495 and GSE62452) was calculated to verify the E3PMP prognostic signature.

### 2.4 Clinical decision analysis

Utilities between E3PMP prognostic signature and AJCC staging were compared using a decision curve analysis (DCA) with “ggDCA” package. A final representative decision tree was learned with the “party” package to visualize the tree structure and resulting decision paths of the E3PMP prognostic signature and clinical characteristics.

### 2.5 Functional cluster analyses

The “limma” package was used to analyze the DEGs between the high risk/expression and the low risk/expression, and gene set enrichment analysis (GSEA) was conducted on Hallmark and Kegg gene sets with the “clusterProfiler” package.

### 2.6 Immune infiltration analyses

The CEBERSORT immune cell enrichment score of TCGA-PAAD was downloaded from the Xena website, and the Pearson correlation coefficient was calculated between the E3PMP score and immune cell enrichment score. The correlation between gene expression and immune cell infiltration in TCGA was queried on the TIMER website.

### 2.7 Protein-protein interaction network analysis

We mapped the differentially expressed genes (DEG) to the Search Tool for the Retrieval of Interacting Genes (STRING) database to identify their potential protein-protein interaction (PPI) relationships at the protein level. The PPI network was visualized using Cytoscape software, and hub genes were selected for further discussion.

### 2.8 Clinical samples

The patients diagnosed with pancreatic cancer by pathological examination were recruited into a pancreatic cancer cohort established in the previous studies. Pancreatic surgeries were performed at the Department of Biliary and Pancreatic Surgery, Tongji Hospital, Tongji Medical College, Huazhong University of Science and Technology, Wuhan, China. Carcinoma samples and paracarcinoma samples were obtained. Informed consent forms were signed before surgery. Postoperatively, patients were followed up to record survival length and adjuvant treatment regimens. All studies involving clinical samples and data were conducted in accordance with the principles of the Helsinki Declaration and were approved by the Ethics Committee of Tongji Hospital, Tongji Medical College, Huazhong University of Science and Technology.

### 2.9 Immunohistochemistry

The pancreatic cancer and paracancerous tissue were fixed with 4% paraformaldehyde and embedded in paraffin. Tissue cores of 1.5 mm that from the 73 tumor tissues and 62 pancreas tissues with complete follow information were used to make tissue the array. WDR37 rabbit polyclonal antibody (Catalog Number: 20916-1-AP, Proteintech, 1:500), CD8 mouse antibody (Catalog Number: 70306S, Cell Signaling Technology, 1:500) and anti-CD68 antibody (Catalog Number: ab283654, abcam, 1:2000) were used in immunohistochemistry. The immunohistochemical staining steps were performed by the standard protocols from the supplier (Maxim EliVision plus polyer HRP Mouse/Rabbit IHC kit KIT-9902). The images were photographed and analyzed on CaseViewer Software version 2.4.

The immunohistochemistry staining results on tissue array were evaluated and quantitatively by two experienced pathologists based on the percentage of positive cells and staining intensity. The percentage score for positive cells ranged from 0 to 4: 0, 0-5%; 1, 6%-25%; 2, 26%-50%; 3, 51%-75%; 4, 76%-100%. Staining intensity was scored on a scale of 0-3: 0, negative; 1, weak; 2, moderate; 3, strong.

### 2.10 Statistical analyses

Continuous variables were summarized as mean ± standard deviation or median (interquartile range), and categorical data are summarized as frequency and percentage. Differences in quantitative data between the WDR37 positive and WDR37 negative groups were compared with student’s t test or the Mann–Whitney U test, and differences in qualitative data were compared with the χ^2^ test or Fisher’s exact test as appropriate. All statistical analyses were performed with the R software v4.2.1 and RStudio, and a *P* value of <0.05 was considered statistically significant.

## 3 Results

### 3.1 Identification of prognostic genes in pancreatic cancer based on E3 ubiquitin ligases

**Figure 1:**
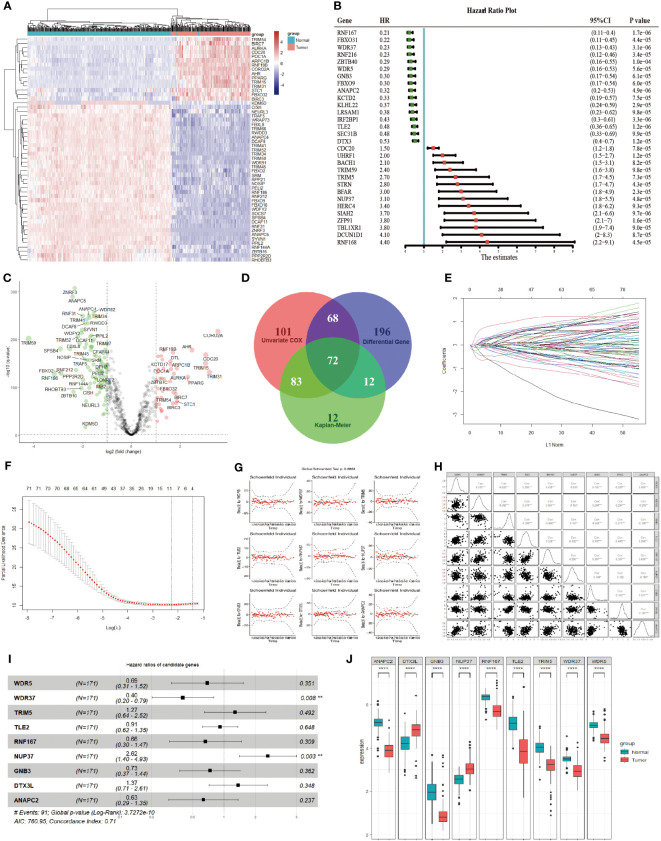
Identification of Prognostic Genes in Pancreatic Cancer Based on E3 Ubiquitin Ligases. **(A)**. The difference in the expression of 898 E3 ubiquitin enzymes between pancreatic cancer (red group) and normal pancreas (blue group). The top 50 differentially expressed genes were presented as expression heatmaps. In the heat map, red indicates upregulation and blue indicates downregulation. **(B)**. The effect of E3 ubiquitin enzyme on overall survival was calculated by log-rank analysis and univariate COX regression analysis. The top 30 genes were showed in the forest map with the lowest P values in univariate COX analysis. Red indicates a risk-associated gene (HR > 1) and green indicates a protective gene (HR < 1). **(C)**. Volcano plots indicate significantly (adjusted *P* value < 0.05) differently expressed genes (DEGs) for E3 ubiquitin enzymes between pancreatic cancer and normal pancreas (above dotted line, Fold change > 1.5). Red indicates upregulation and blue indicates that the gene is downregulated. **(D)**. Venn gram was used to screen genes with statistical significance (*P* < 0.05) in univariate COX regression analysis, log-rank survival analysis and difference analysis. A total of 72 candidate genes were identified for the next analysis. **(E)**. LASSO coefficient profiles of the prognostic genes in LASSO-COX regression. **(F)**. Parameter selection in LASSO model. **(G)**. Results of hypothesis test of the candidate genes in multivariate COX regression analysis. **(H)**. Collinearity analysis between the candidate genes, and collinearity analysis between the candidate genes. **(I)**. Forest plot of the candidate genes in multivariate COX regression analysis. The prognosis risk of candidate genes in the multivariable Cox model were calculated, and a significance level of *P* < 0.05 was considered statistically significant. **(J)**. Expression of the candidate genes between pancreatic cancer (red group) and normal pancreas (blue group) according to TCGA and GTEx. The expression differences of candidate genes between two groups were assessed using the t-test, where a significance level of *P* < 0.05 was considered statistically significant. *P < 0.05; **P < 0.01; ***P < 0.001; ****P < 0.0001.

The difference of 898 E3 ubiquitin enzymes from IUUCD database were analyzed between pancreatic cancer in TCGA and normal pancreatic tissues in GTEx database, and 348 DEGs were identified (**Figures 1A, B**). Univariate Cox regression and log-rank survival analysis were performed, and 324 and 179 E3 ubiquitin enzymes were found to be associated with overall survival of pancreatic cancer patients respectively (**Figure 1C**). As a result, 72 DEGs related to overall survival were identified in the synthesis of survival analyses and differential analyses (**Figure 1D**). Subsequently, 72 survival-related differential E3 ubiquitin enzymes were input into the LASSO-Cox regression model for further selection (**Figures 1E, F**). As a result, 9 candidate genes (WDR5, WDR37, TRIM5, TLE2, RNF167, NUP37, GNB3, DTX3L, ANAPC2) were identified, which had been assessed by collinearity and hypothesis tests (**Figures 1G, H**). A multivariate Cox proportional hazards regression model constructed the 9 genes as a prognostic signature for overall survival, and the formula was as follows: risk score = (0.965 × NUP37 exp) + (0.311 × DTX3L exp) + (0.240 × TRIM5 exp) + (-0.091 × TLE2 exp) + (-0.315 × GNB3 exp) + (-0.378 × WDR5 exp) + (-0.415 × RNF167 exp) + (-0.462 × ANAPC2 exp) + (-0.918 × WDR37 exp) (**Figure 1I**). Notably, the only independent protective factor WDR37 was low expression in pancreatic cancer, and the only independent risk factor NUP37 was high expression in pancreatic cancer (**Figure 1J**).

### 3.2 Utilizing the E3PMP prognostic signature for accurate prediction of pancreatic cancer prognosis

**Figure 2:**
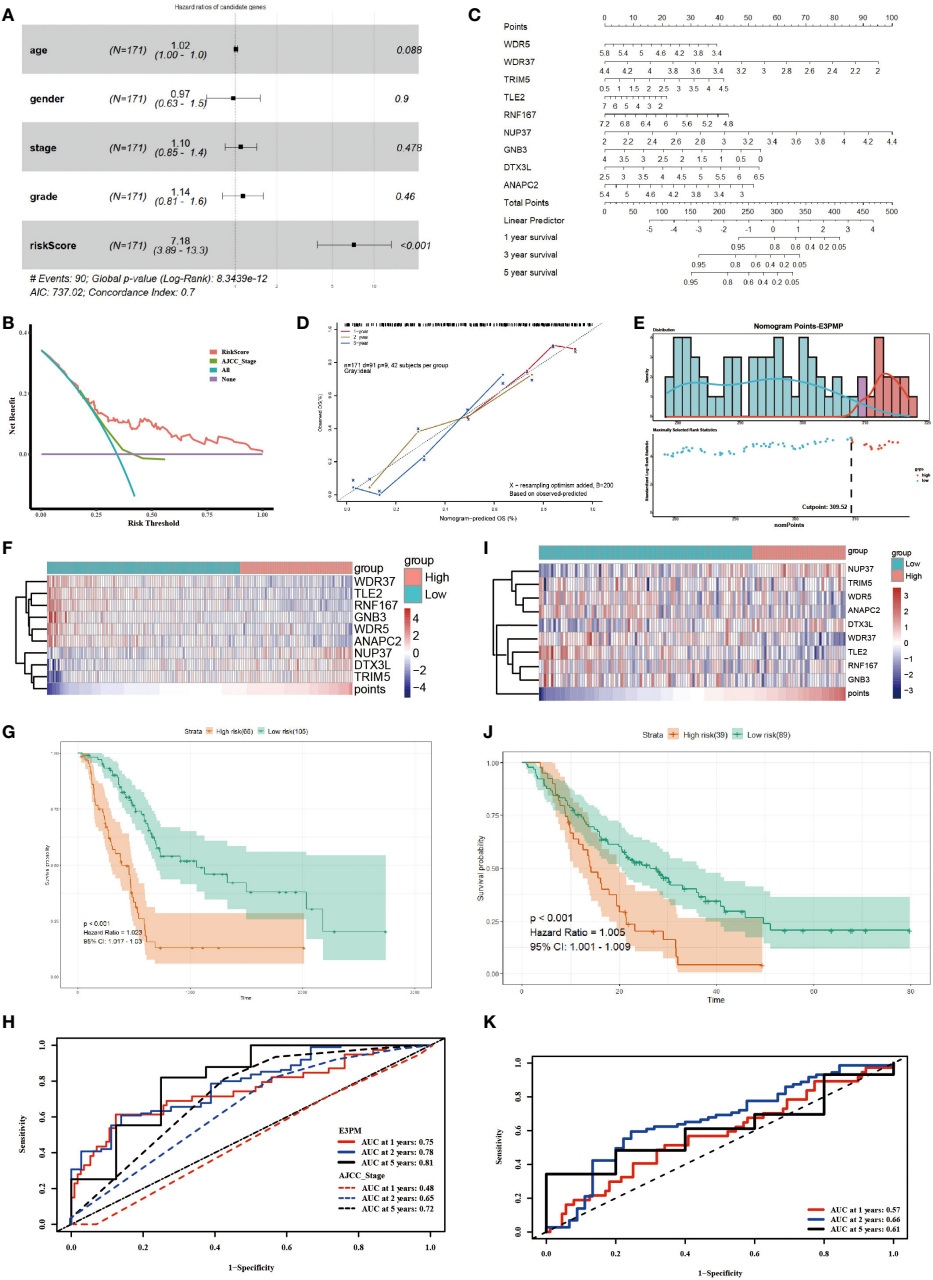
Utilizing the E3PMP Prognostic Signature for Accurate Prediction of Pancreatic Cancer Prognosis. **(A)**. Forest plot of multivariate COX regression analysis of TCGA clinical characteristics and the risk score of candidate genes. **(B)**. Clinical usefulness of risk score and AJCC staging system were assessed by decision curve analysis. Red indicates the risk score of multivariate COX regression model and green indicates the AJCC staging system as a control. **(C)**. Nomogram for predicting 1-, 2-, 5-year overall survival of pancreatic cancer patient**s** in the TCGA dataset according to the multivariate COX regression model. **(D)**. The calibration curve of the nomogram for predicting 1-(red), 2-(yellow), 5-(blue) year overall survival of the pancreatic cancer patients in the TCGA dataset. **(E)**. The distribution of survival status and E3PMP score, which indicates the cutoff point of E3PMP score. **(F)**. Heat map of prognostic genes expression in the TCGA dataset. **(G)**. Overall survival analysis of patients in the TCGA dataset between the high- and low-E3PMP groups. The survival differences between high-risk and low-risk groups were evaluated using the log-rank test, with a significance level of *P* < 0.05 indicating statistical significance. **(H)**. Time-dependent receiver operating characteristic curves of the E3PMP signature for predicting 1-, 2-, and 5-year overall survival of the pancreatic cancer patients in the TCGA dataset. **(I)**. Heat map of prognostic genes expression in the merged GEO (GSE57495 and GSE62452) dataset as a validation. **(J)**. Overall survival analysis of patients in the merged GEO (GSE57495 and GSE62452) dataset between the high-E3PMP and low-E3PMP groups as a validation. **(K)**. Time-dependent receiver operating characteristic curves of the E3PMP signature for predicting 1-, 2-, and 5-year overall survival of the pancreatic cancer patients in the merged GEO (GSE57495 and GSE62452) dataset as a validation.

According to the formula, the risk score of each sample in TCGA-PAAD was calculated. Furthermore, the risk score was indicated to be an independent prognostic factor for survival of the patients, comparing with the clinical characteristics (**Figure 2A**). The decision curve analysis (DCA) confirmed that the risk score was more clinically applicable than that of the traditional AJCC staging system (**Figure 2B**). The results demonstrated that the nine E3 ubiquitin enzymes can accurately predict the prognosis of pancreatic cancer patients.

A nomogram of E3PMP (E3 enzymes prognostic model of pancreatic cancer) prognostic signature was established based on the results of the multivariate Cox regression to predict the overall survival of pancreatic cancer patients (**Figure 2C**). A calibration plot was used to evaluate the precision of predicting the 1-, 2-, and 5-year overall survival, all of which exhibited strong correlation with the nomogram findings (**Figure 2D**). An optimal cut-off (E3PMP score =309.52) was used as grouping criteria for high risk and low risk groups (**Figure 2E**). With the increase of E3PMP score, the expression of risk genes (TRIM5, NUP37, DTX3L) increased, while the expression of protective genes (WDR5, WDR37, TLE2, RNF167, GNB3, ANAPC2) decreased (**Figure 2F**). Survival analysis showed that patients with higher E3PMP scores had a higher risk of death, whose overall survival was significantly worse than that of patients with low E3PMP scores (**Figure 2G**). The time-dependent ROC curve and its AUC value of 1-year, 2-year and 5-year overall survival were analyzed to evaluate the prognostic significance of this stratification, indicating that the E3PMP prognostic signature might be more clinically applicable than traditional AJCC staging (0.75, 0.78, 0.81 and 0.48, 0.65, 0.72, respectively) (**Figure 2H**).

Using the same methods, the merged dataset of GSE57495 and GSE62452 from GEO database was analyzed as the verification set to verify the E3PMP prognostic signature. The cut-off value of the E3PMP score in the training set (TCGA) was used as grouping criteria in the verification set (GEO). Similarly, risk genes (TRIM5, NUP37, DTX3L) showed a trend of high expression in the high risk group, while protective genes (WDR5, WDR37, TLE2, RNF167, GNB3, ANAPC2) showed a high expression trend in the low risk group (**Figure 2I**). The survival of patients with high risk of E3PMP score was significantly worse than that of patients with low risk (**Figure 2J**). The predictive value of the E3PMP prognostic signature was verified by time-dependent ROC curve, and the AUC values of 1-year, 3-year and 5-year were 0.57,0.66 and 0.61 respectively (**Figure 2K**).

These results suggest that the E3PMP prognostic signature can be used to predict the survival of patients with pancreatic cancer.

### 3.3 Predicting immunocyte infiltration in pancreatic cancer using the E3PMP prognostic signature

**Figure 3:**
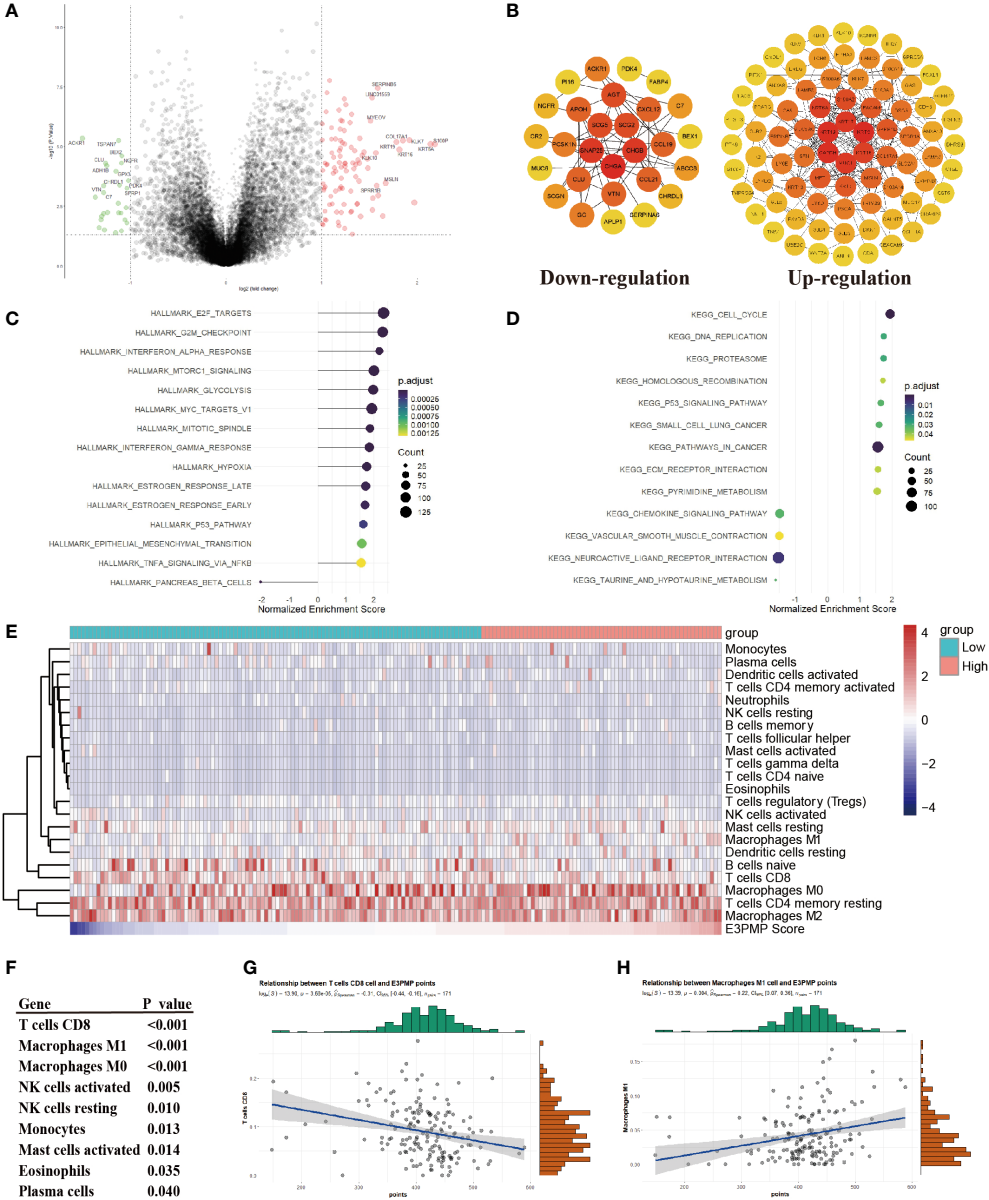
Predicting Immunocyte Infiltration in Pancreatic Cancer Using the E3PMP Prognostic Signature. **(A)**. Volcano plots indicate significantly (adjusted *P* value < 0.05) differently expressed genes (DEGs) between the high- and low-E3PMP groups (above dotted line, Fold change > 1). Red indicates upregulation and blue indicates that the gene is downregulated. **(B)**. PPI network indicates down-regulated and up-regulated differently expressed genes between the high- and low-E3PMP groups. **(C)**. Gene Set Enrichment Analysis (GSEA) of differentially expressed genes between the high- and low-E3PMP groups using the HALLMARK gene set indicates the top 15 significant enriched signal pathways. **(D)**. Gene Set Enrichment Analysis (GSEA) of differentially expressed genes between the high- and low-E3PMP groups using the KEGG gene set indicates the top 15 significant enriched signal pathways. **(E)**. The heat map indicates the difference of CIBORSORT immune cell enrichment score between the high-E3PMP and low-E3PMP groups. **(F)**. The significantly (person analysis, *P* value < 0.05) immune cells indicating the correlations of CIBORSORT immune cell enrichment score with the E3PMP score. **(G)**. The correlations of CIBORSORT immune cell enrichment score of CD8 positive T cells with the E3PMP score. **(H)**. The correlations of CIBORSORT immune cell enrichment score of M1 macrophages with the E3PMP score.

In order to explore the mechanism of the effect on the survival, the differential genes between high risk group and low risk group were analyzed (**Figure 3A**). The PPI network of DEGs was constructed by STRING, which suggested that the main hub genes of high expression genes included important oncogenes such as MUC1, KRT16, KRT5, KRT17 and KRT19, while the main hub genes of high expression genes in low risk group included tumor suppressor genes such as CHGA, CNAP25, CHGB, SCG2 and SCG5 (**Figure 3B**). Meanwhile, the GSEA analyses of the DEGs comparing the groups between high and low E3PMP scores showed that cancer promoting pathways such as E2F, G2M checkpoint, INF-α, mTORC1 and cell cycle signals were significantly up-regulated, while tumor suppressor pathways such as primary immune response, chemokine receptor and immune rejection were significantly down-regulated (**Figures 3C, D**).

Considering that the immunology related pathways were down-regulated in the group of high E3PMP score, the CIBORSORT immune infiltration data of TCGA-PAAD were used to evaluate whether E3PMP signature was correlated with the immune cell infiltration (**Figure 3E**). As a result, E3PMP score was associated with infiltration of CD8 positive T cells, M1 macrophages, M0 macrophages, activated or resting NK cells, monocytes, activated mast cells, eosinophils and plasma cells (**Figure 3F**). Notably, CD8 positive T cells and M1 macrophages showed the strongest correlation with E3PMP score. Tumor suppressing CD8 positive T cells were negatively correlated with E3PMP score (**Figure 3G**), and cancer promoting M1 macrophages were positively correlated with E3PMP score (**Figure 3H**).

### 3.4 Identification of WDR37 as the leading gene in the E3PMP prognostic signature

**Figure 4:**
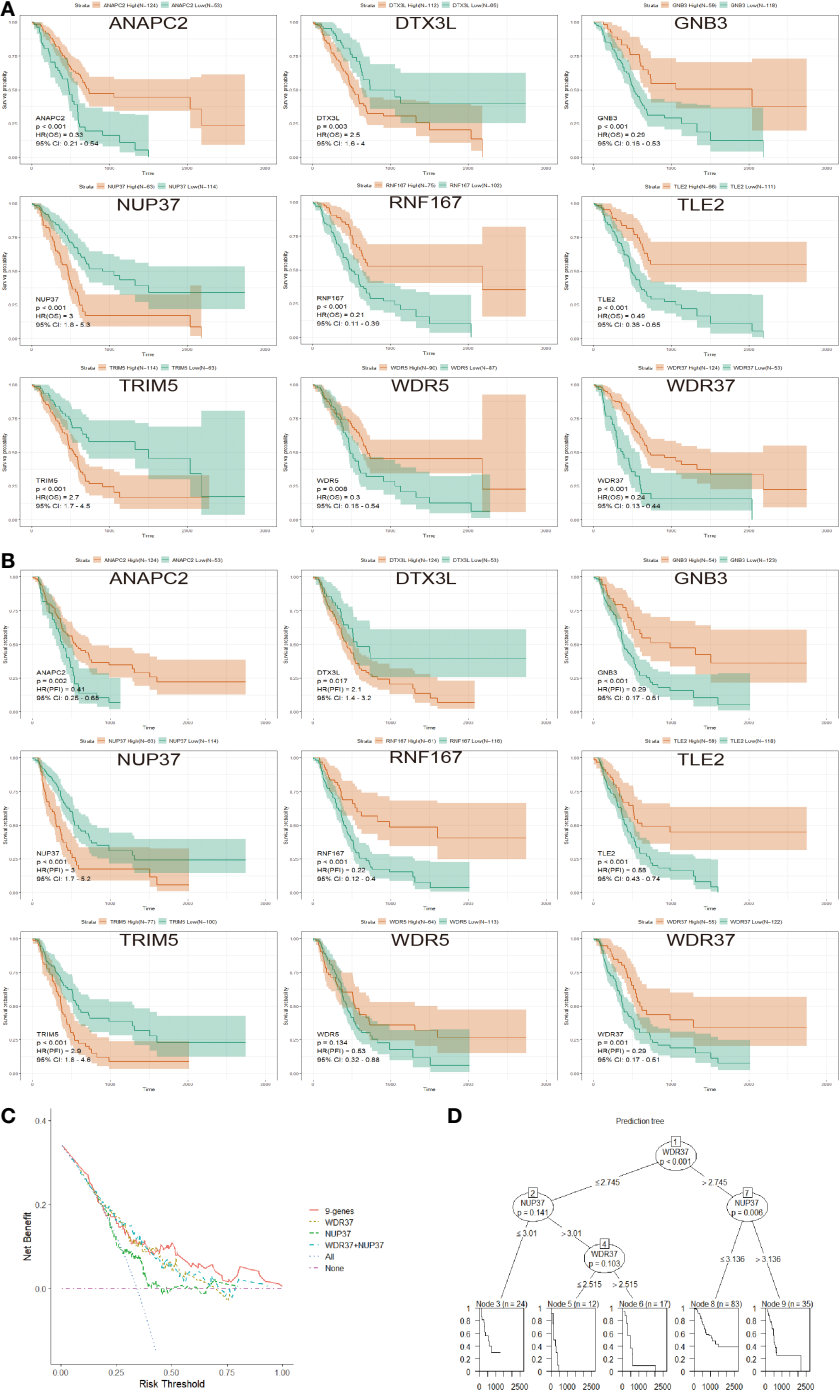
Identification of WDR37 as the Leading Gene in the E3PMP Prognostic Signature. **(A)**. The overall survival of the patients in TCGA dataset between the groups with the high- and low- expression of candidate genes. **(B)**. The progression free interval of the patients in TCGA dataset between the groups with the high- and low- expression of candidate genes. **(C)**. Compression of the clinical usefulness among the E3PMP score, WDR37 expression, NUP37 expression and WDR37+NUP37 expression were assessed by decision curve analysis. **(D)**. Decision tree was assessed to predict the priority of the candidate genes in the E3PMP prognostic signature.

In order to compare the influence of each candidate genes on survival in E3PMP prognostic signature, the overall survival and private finance initiative survival were further evaluated (**Figures 4A, B**). As a result, NUP37 was the most significant oncogene, while WDR37 was the most significant tumor suppressor gene. Furthermore, the clinical predictive value of E3PMP signature (nine genes model), WDR37 expression, NUP37 expression and two genes model (WDR37 and NUP37) were compared with DCA (**Figure 4C**). The results showed that the predictive value of WDR37 expression was close to that of two-gene and nine-gene models, and significantly better than NUP37. Meanwhile, the clinical decision tree was constructed according to the E3PMP prognostic signature, showing that the expression of WDR37 was the first node in the decision tree (**Figure 4D**). These results suggest that WDR37 was the decisive gene in E3PMP prognostic signature and may be the most valuable E3 ubiquitin enzyme in evaluating the prognosis of pancreatic cancer.

### 3.5 The association between WDR37-mediated immunocyte infiltration and prognosis in pancreatic cancer

**Figure 5:**
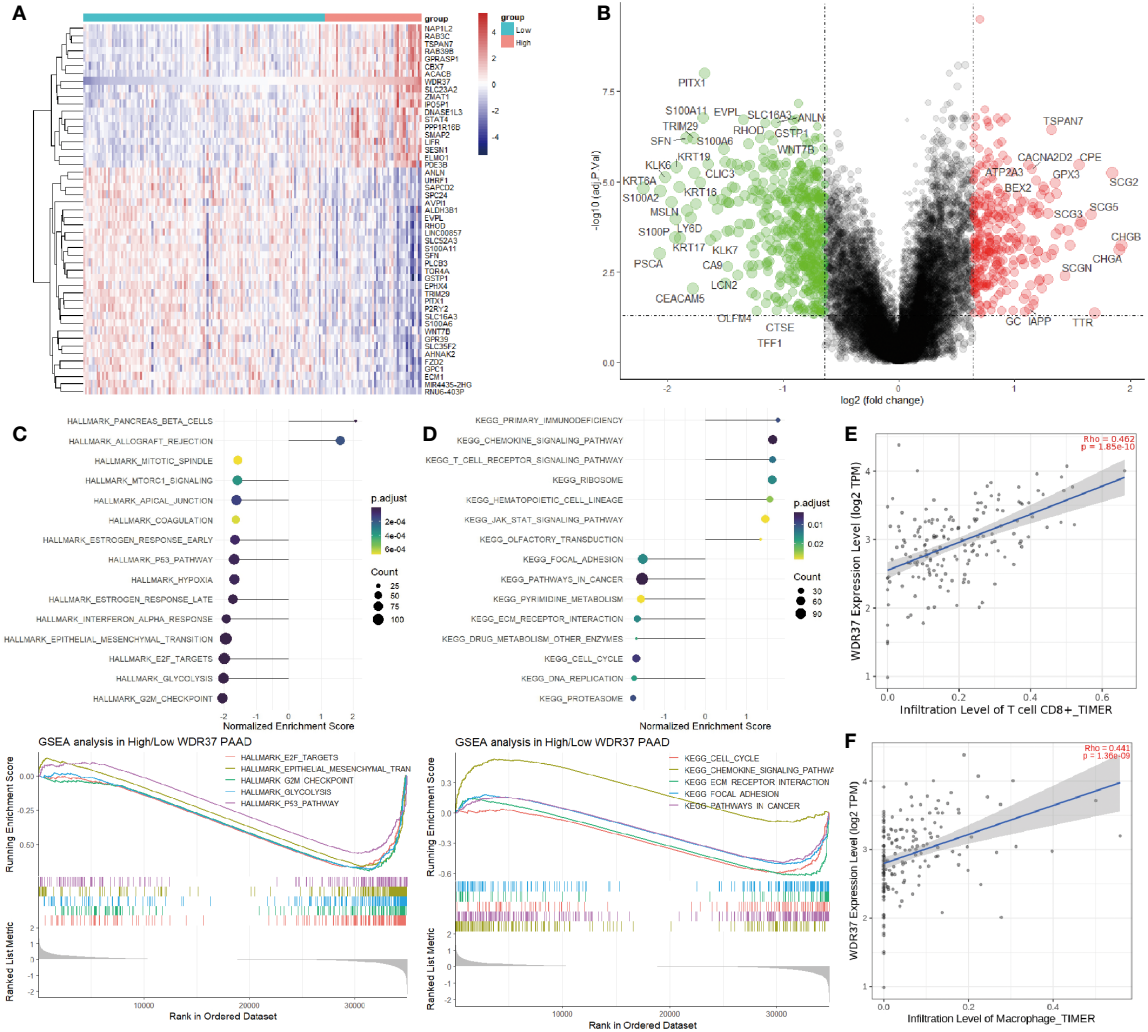
The Association Between WDR37-Mediated Immunocyte Infiltration and Prognosis in Pancreatic Cancer **(A)**. The top 50 differentially expressed genes were presented as expression heatmaps comparing the difference between the high- and low- WDR37 expression groups. In the heat map, red indicates upregulation and blue indicates downregulation. **(B)**. Volcano plots indicate significantly (adjusted *P* value < 0.05) differently expressed genes (DEGs) between the high- and low- WDR37 expression groups (above dotted line, Fold change > 0.6). Red indicates upregulation and blue indicates that the gene is downregulated. **(C)**. Gene Set Enrichment Analysis (GSEA) of differentially expressed genes between the high- and low-WDR37 expression groups using the HALLMARK gene set indicates the top 15 significant enriched signal pathways. The GSEA curves are presented to emphasize the important pathways in pancreatic cancer. **(D)**. Gene Set Enrichment Analysis (GSEA) of differentially expressed genes between the high- and low-WDR37 expression groups using the KEGG gene set indicates the top 15 significant enriched signal pathways. The GSEA curves are presented to emphasize the important pathways in pancreatic cancer. **(E)**. The correlations of immune cell enrichment score of CD8 positive T cells with the WDR37 expression. **(F)**. The correlations of immune cell enrichment score of macrophages with the WDR37 expression.

The function of WDR37 needs to be analyzed to explore how it affects the prognosis of the patients. An optimal cut-off of expression was used as grouping criteria for high WDR37 and low WDR37 groups to assess the DEGs (**Figures 5A, B**). GSEA analyses showed that the high expression of WDR37 was related to the activation of tumor suppressor pathways such as primary immunity, immune rejection, and T cell signal transduction, while the low expression of WDR37 was related to the activation of G2M checkpoint, glycolysis, cell cycle and EMT (**Figures 5C, D**). Furthermore, the effect of WDR37 expression on immune infiltration was evaluated by TIMER database, and it was found that the high expression of WDR37 was related to the increase the infiltration of CD8 positive T cells and macrophages (**Figures 5E, F**).

In order to explore the potential mechanism of the effect of WDR37 on the prognosis of pancreatic cancer, WDR37 was overexpressed in pancreatic cancer cell line SW1990 to identify the binding proteins by immunoprecipitation-mass spectrometry (**Supplementary Data**). The immunoprecipitation-mass spectrometry indicated that there may be a strong interaction between WDR37 and TCP1 complex (TCP1, CCT4, CCT8, CCT6A, CCT5, CCT7) (**Supplementary Data**). At the same time, mass spectrometry showed that there was a binding of K48 ubiquitin (UBA52) chain on WDR37 (**Supplementary Data**). In summary, WDR37 may play an anti-tumor role by mediating the degradation of TCP1 complex through K48 ubiquitin modification.

### 3.6 The value of WDR37 in evaluating clinical prognosis of pancreatic cancer

**Figure 6:**
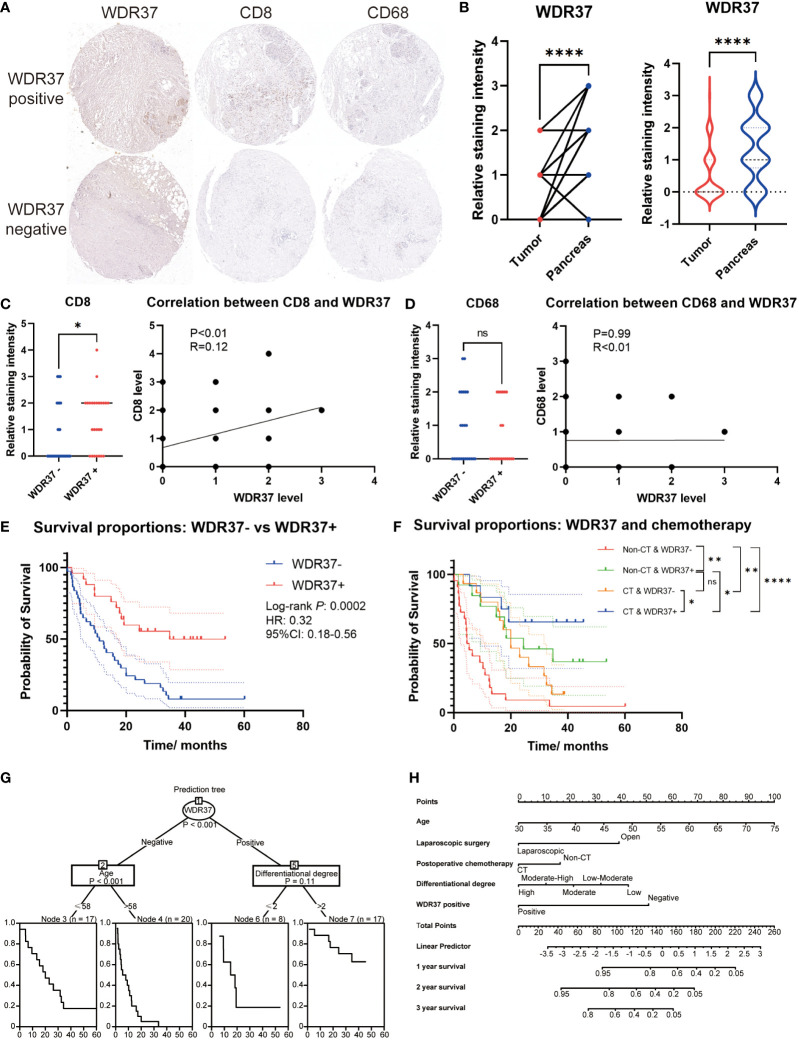
The Value of WDR37 in Evaluating Clinical Prognosis of Pancreatic Cancer **(A)**. Immunohistochemical staining of WDR37, CD8 and CD68 between paracancerous tissue and tumor tissue in the pancreatic cancer. **(B)**. The difference of relative WDR37 staining intensity between tumor tissues and paracancerous tissues, comparing with paired t-test (left) and unpaired t-test (right). **(C)**. The difference of relative CD8 staining intensity between samples with WDR37 negative and samples with WDR37 positive, and the correlation between the expression level of CD8 and WDR37. **(D)**. The difference of relative CD68 staining intensity between samples with WDR37 negative and samples with WDR37 positive, and the correlation between the expression level of CD68 and WDR37. **(E)**. The overall survival of the patients in tissue array between the positive-WDR37 (blue) and negative-WDR37 (red) groups. **(F)**. The overall survival of the patients in tissue array considering the chemotherapy and WDR37 expression. Red indicates the patients with negative WDR37 and non- chemotherapy; green indicates the patients with positive WDR37 and non- chemotherapy; yellow indicates the patients with negative WDR37 and chemotherapy; blue indicates the patients with positive WDR37 and chemotherapy. **(G)**. Decision tree was assessed to predict the priority among the WDR37 expression, age, postoperative chemotherapy, differentiational degree and surgical approach. **(H)**. Nomogram for predicting 1-, 2-, 3-year overall survival of pancreatic cancer patients considering the WDR37 expression, age, postoperative chemotherapy, differentiational degree and surgical approach. *P < 0.05; **P < 0.01; ***P < 0.001; ****P < 0.0001.

The tissue array of clinical samples was assessed to verify the expression characteristics and prognosis effect of WDR37 on pancreatic cancer patients (**Figure 6A**). In the clinical cohort, it was comparable in the basic characteristics, pathological classification, preoperative CA19-9 level and postoperative chemotherapy between the WDR37 negative patients and WDR37 positive patients (**Table 1**). Immunohistochemistry showed that the expression of WDR37 in pancreatic cancer tissues was significantly lower than that in paracancerous tissues, and the expression of WDR37 in normal pancreatic tissues was significantly higher than that in pancreatic cancer tissues (**Figure 6B**). The statistical analysis of the tissue chips revealed a higher presence of CD8 positive T lymphocyte infiltration in WDR37 positive samples (**Figure 6C**). In contrast, the expression of CD68 was found to be unrelated to WDR37 expression (**Figure 6D**).

**Table 1:** Characteristics of patients in the tissue array.

	WDR37 negative (N=37)	WDR37 positive (N=25)	*P*
Age [mean (SD)]	58.49 (9.95)	57.40 (8.19)	0.653
Smoking (%)	9 (24.3)	6 (24.0)	>0.999
Drinking (%)	7 (18.9)	4 (16.0)	>0.999
Diabetes (%)	4 (10.8)	3 (12.0)	>0.999
Hepatitis (%)	1 (2.7)	3 (12.0)	0.350
CA19-9 (median [IQR])	173.50 [19.85, 682.70]	75.31 [33.45, 719.30]	0.693
Laparoscopic surgery (%)	19 (51.4)	14 (56.0)	0.920
Postoperative chemotherapy (%)	15 (40.5)	12 (48.0)	0.749
R1 resection (%)	7 (18.9)	2 (8.0)	0.407
Nervous invasion (%)	7 (18.9)	6 (24.0)	0.870
Vascular invasion (%)	5 (13.5)	3 (12.5)	>0.999
Differentiational degree (%)			0.504
Low	7 (18.9)	3 (12.0)	
Low-Moderate	13 (35.1)	5 (20.0)	
Moderate	13 (35.1)	12 (48.0)	
Moderate-High	3 (8.1)	3 (12.0)	
High	1 (2.7)	2 (8.0)	
Tumor location (%)			0.458
Head	22 (59.5)	18 (72.0)	
Body-Tail	15 (40.5)	7 (28.0)	
AJCC-T stage (%)			0.179
T1	4 (10.8)	4 (16.0)	
T2	21 (56.8)	18 (72.0)	
T3	12 (32.4)	3 (12.0)	
AJCC-N stage (%)			0.664
N0	20 (54.1)	16 (64.0)	
N1	16 (43.2)	8 (32.0)	
N2	1 (2.7)	1 (4.0)	
AJCC-M stage (%)			0.842
M0	37 (100.0)	24 (96.0)	
M1	0 (0.0)	1 (4.0)	
AJCC stage (%)			0.245
Ia	2 (5.4)	3 (12.0)	
Ib	14 (37.8)	13 (52.0)	
IIa	4 (10.8)	0 (0.0)	
IIb	16 (43.2)	7 (28.0)	
III	1 (2.7)	1 (4.0)	
IV	0 (0.0)	1 (4.0)	
OS time (median [IQR])	10.97 [4.43, 20.03]	25.07 [16.70, 39.70]	0.002
CD8 positive	12 (32.4)	18 (72.0)	0.005
CD68 positive	11 (45.8)	8 (38.1)	0.824

Survival analysis showed that the overall survival of WDR37 positive patients was significantly better than WDR37 negative patients (**Figure 6E**). Interestingly, the overall survival of WDR37 positive patients was no worse than WDR37 negative patients, regardless of the application of postoperative chemotherapy (**Figure 6F**). Univariate COX regression analysis indicated that positive WDR37, higher differentiational degree, application of laparoscopic surgery and receiving postoperative chemotherapy were independent protective factors for survival of patients with pancreatic cancer, while the elder age was the independent risk factor (**Table 2**).

**Table 2:** COX regression analyses of overall survival.

Variable	Univariate COX regression			Multivariate COX regression		
	HR	95% CI	*P*	HR	95% CI	*P*
Age	1.07	(1.03-1.11)	<0.001	1.07	(1.02-1.12)	0.002
Smoking	1.09	(0.56-2.11)	0.794			
Drinking	1.04	(0.48-2.23)	0.928			
Diabetes	1.01	(0.40-2.59)	0.981			
Hepatitis	0.57	(0.14-2.36)	0.436			
CA19-9	1.00	(1.00-1.02)	0.064			
Laparoscopic surgery	0.39	(0.22-0.72)	0.002	0.31	(0.16-0.60)	<0.001
Postoperative chemotherapy	0.46	(0.25-0.85)	0.013	0.61	(0.31-1.22)	0.163
R1 resection	1.93	(0.92-4.04)	0.083			
Nervous invasion	0.60	(0.27-1.35)	0.219			
Vascular invasion	0.71	(0.28-1.81)	0.476			
Differentiational degree	0.64	(0.48-0.85)	0.002	0.72	(0.53-1.00)	0.048
Tumor location	1.42	(0.78-2.57)	0.248			
AJCC-T stage	1.55	(0.95-2.52)	0.077			
AJCC-N stage	1.36	(0.85-2.17)	0.203			
AJCC-M stage	1.17	(0.16-8.55)	0.878			
AJCC stage	1.23	(0.98-1.54)	0.074			
WDR37 positive	0.30	(0.16-0.59)	<0.001	0.22	(0.11-0.45)	<0.001
CD8 positive	0.82	(0.46-1.48)	0.516			
CD68 positive	1.16	(0.58-2.33)	0.668			

Clinical decision tree analysis showed that the WDR37 expression was the most important factor for predicting the prognosis of the patients (**Figure 6G**). Finally, a nomogram for predicting the prognosis of pancreatic cancer patients was constructed according to multivariate Cox regression model to facilitate clinical practice (**Figure 6H**). These results suggested that WDR37 is an important protective factor affecting the prognosis of patients with pancreatic cancer.

## 4 Discussion

In this study, we developed an E3 ligase-based signature of 9 genes, including TRIM5, NUP37, DTX3L, WDR5, WDR37, TLE2, RNF167, GNB3, and ANAPC2. In the training set of TCGA and the verification set of GEO database, the signature was proved to accurately predict the prognosis of patients with pancreatic cancer. Furthermore, functional cluster analysis and clinical decision analysis suggested that WDR37 might be the most prognostic E3 ubiquitin ligase in pancreatic cancer. The results of functional analysis, *in vitro* experiments and clinical cohort studies suggested a tumor‐suppressive role of WDR37, and the mechanism may be related to the role of TCP1 complex.

Among the nine genes in the prognostic signature, some studies have confirmed that they may be related to tumor progression. TRIM5 has been reported as a prognostic factor for hepatocellular carcinoma and glioma, which may affect the immune and inflammatory response of virus-infected cells by promoting Lys-63 ubiquitination of MAP3K7/TAK1 complex, but the exact role of TRIM5 in tumors has not been reported (13, 14). NUP37 is a major component of the nuclear pore complex, which regulates the stability of YAP1 in a LRP5-dependent manner and promotes the progression of hepatocellular carcinoma (15). DTX3L is reported to regulate the ubiquitin modification of PARP family proteins (PARP1, PARP2, PARP9, PARP14), promoting proliferation, migration and chemotherapy resistance of lymphoma, glioma, and melanoma (16). Studies have shown that WDR5 acts as the core scaffold component of histone methyltransferase complex, which promote the progression and drug resistance of many kinds of tumors (17). TLE2 has been reported for many times to inhibit tumor progression and resistance to gemcitabine in pancreatic cancer, but the specific molecular mechanism is not clear (18, 19). RNF167 inhibited the activity of mTORC1 by regulating the Lys-63-linked and Lys-29-linked ubiquitination of SESN2 and CASTOR1, which played a significant tumor-suppressive role in colon and breast cancer (20, 21). The gene polymorphism of GNB3 is related to the survival of many kinds of tumors, but its specific molecular mechanism is not clear (22). ANAPC2 was upregulated by artesunate and degraded the KRAS protein in colon cancer cells, so ANAPC2 may play an important role in pancreatic cancer in a similar manner (23).

Much less is known about the role of WDR37 in tumors. WDR37 has been reported to interact with PACS1, thus regulating the release of Ca^2+^ from endoplasmic reticulum in lymphocytes and the function of immune cells (24). Our study suggested that WDR37 might promote the degradation of TCP1 complex, which has never been reported in previous studies. The TCP1 complex consists of eight homologous subunits (TCP1, CCT2, CCT3, CCT4, CCT5, CCT6, CCT7, CCT8), which regulate telomere maintenance and protein folding (25). Systematic studies in hepatocellular carcinoma and breast cancer have demonstrated that CCT proteins were associated with immune cell infiltration and tumor progression (26, 27). The role of CCT protein in pancreatic cancer was not clear, while CCT6A was reported to promote TGF-β signal transduction in prostate cancer, which may promote fibrosis in pancreatic cancer (28). TCP1 protein promoted the activation of PI3K/AKT1/mTORC1 signal in ovarian cancer, supposing that TCP1 played a tumor-promoting role in pancreatic cancer (29). In conclusion, WDR37 may inhibit the progression of pancreatic cancer and promote tumor immune response by promoting the degradation of TCP1 protein in pancreatic cancer.

It should be noted that our E3PMP signature is more instructive than other types of prognostic signatures in previous studies, because E3 ubiquitin ligase has a strong potential for drug development. WDR37 might be an important tumor suppressor gene in pancreatic cancer and the possible mechanism was revealed *in vitro*. The results of the clinical cohort further confirmed the strong prognostic value of WDR37, because the overall survival of WDR37-positive pancreatic cancer patients without postoperative chemotherapy was significantly better than that of WDR37-negative pancreatic cancer patients with postoperative chemotherapy. However, this study also has some shortcomings, some potentially more meaningful prognostic genes were dismissed in this study, because only the E3 ubiquitin ligases were analyzed. In addition, although this study suggested that WDR37 was a strong tumor suppressor for pancreatic cancer, further studies are needed to reveal its specific molecular mechanism.

In conclusion, the E3PMP signature accurately predicted the prognosis of patients with pancreatic cancer, so it can be used as a decision-making tool to guide the treatment of patients with pancreatic cancer. At the same time, WDR37, the main gene in E3PMP signature, can be used as the most prognostic E3 ubiquitin ligase in the treatment of pancreatic cancer.

## Data availability statement

The datasets presented in this study can be found in online repositories. The database names and the accession numbers can be found in the methods section of the article. For clinical data and code, please contact Qin R (ryqin@tjh.tjmu.edu.cn) for access.

## Ethics statement

This study was approved by the Institutional Review Board of Tongji Medical College, Huazhong Scientific and Technological University. The studies were conducted in accordance with the local legislation and institutional requirements. The participants provided their written informed consent to participate in this study.

## Author contributions

TY: Conceptualization, Data curation, Formal analysis, Funding acquisition, Investigation, Methodology, Project administration, Resources, Software, Validation, Visualization, Writing – original draft, Writing – review & editing. JW: Conceptualization, Data curation, Funding acquisition, Resources, Visualization, Writing – original draft. SX: Data curation, Formal analysis, Resources, Writing – original draft. LC: Validation, Writing – review & editing. ZZ: Investigation, Methodology, Writing – review & editing. SP: Validation, Writing – review & editing. MZ: Investigation, Writing – review & editing. XG: Supervision, Writing – review & editing. MW: Funding acquisition, Project administration, Writing – review & editing. JG: Funding acquisition, Project administration, Resources, Supervision, Writing – review & editing. HZ: Project administration, Resources, Supervision, Writing – review & editing. RQ: Conceptualization, Funding acquisition, Project administration, Resources, Writing – review & editing.
